# Impulsivity, internalizing symptoms, and online group behavior as determinants of online hate

**DOI:** 10.1371/journal.pone.0231052

**Published:** 2020-04-22

**Authors:** Markus Kaakinen, Anu Sirola, Iina Savolainen, Atte Oksanen

**Affiliations:** 1 Institute of Criminology and Legal Policy, University of Helsinki, Helsinki, Finland; 2 Faculty of Social Sciences, Tampere University, Tampere, Finland; Valparaiso University, UNITED STATES

## Abstract

Online hate is widely identified as a social problem, but its social psychological dimensions are yet to be explored. We used an integrative social psychological framework for analyzing online hate offending and found that both personal risk factors and online group behavior were associated with online hate offending. Study 1, based on socio-demographically balanced survey data (*N* = 1200) collected from Finnish adolescents and young adults, found that impulsivity and internalizing symptoms were positively associated with online hate offending. Furthermore, social homophily was positively associated with online hate offending but only among those with average or high level of internalizing symptoms. Social identification with online communities was not associated with hate offending. In Study 2, based on a vignette experiment (*N* = 160), online hate offenders were more likely than others to rely on in-group stereotypes (i.e. self-stereotype) in anonymous online interaction and, as a consequence, follow perceived group norms. These associations were found only when a shared group identity was primed. We conclude that both personal risk factors and group behavior are related to online hate but they have different implications for reducing hateful communication in social media.

## Introduction

As human communication is increasingly embedded in social media platforms, hostile online behavior has become an apparent social problem [1, 2]. One form of aggressive behavior is online hate (i.e., cyberhate) that involves behavior that offends or threatens other individuals or social groups [3, 4, 5]. Viewing hateful online content has become a frequent experience among young people [6, 7]. Several qualities make social media a particularly suitable environment for expressing hate including anonymity [8, 9] and lack of surveillance and control [10, 11, 12]. Hate is also a powerful driver of social interaction and participation online [2, 13, 14].

Explanations for antisocial online behavior include personal risk factors for online offending such as impulsivity [15, 16] and mental health problems [17, 18]. Antisocial behavior on the Internet is also related to online group behavior that motivates hostilities between different identity-based groups. Especially, social identification and deindividuation are suggested as a psychological mechanism of online hostilities [19, 20, 21]. Social homophily and polarization between different online communities have also been reported to motivate aggression in social media [22, 23].

In recent literature, integrative models including personal and situational risk factors are suggested for explaining violent and criminal behavior [24, 25, 26] and online aggression [27]. In integrated models, different determinants of antisocial behavior are combined to reach more comprehensive conclusions. This approach is at the core of social psychological inquiry [28, 29].

In this study, we used the integrative approach including impulsivity and internalizing symptoms as personal risk factors and online group behavior (i.e. homophily, social identification and self-stereotyping) to analyze online hate offending among adolescents and young adults. The research focusing on online aggression has increased in recent years [27], but this is the first attempt to analyze online hate offending from an integrative social psychological framework. Our focus was on adolescents and young adults, as they are active and engaged users of different online communication platforms [30, 31].

### Impulsivity, internalizing symptoms, and online aggression

Impulsivity is a multidimensional concept characterized by a tendency to engage in maladaptive behavior and the inability to control one’s thoughts and behavior [32, 33]. As a facet of personality, impulsivity is characterized by urgency, lack of premeditation, lack of perseverance, and sensation seeking [34]. Impulsivity is considered as a core component of many personality disorders, such as antisocial and borderline personality disorders, as defined in the fifth revision of the Diagnostic and Statistical Manual of Mental Disorders (DSM-V) [35]. In online interaction, many features such as anonymity and reduced social presence might make impulsive individuals less likely to self-reflect or hesitate before posting hostile content [11]. High impulsiveness is associated with aggressive behavior in general [36] and in online environments [15, 16].

Internalizing symptoms relate to negative-affect-laden disorders such as depression or anxiety [37, 38]. Intensive negative affective states can lead to dysfunctional emotional and behavioral regulation [39], and internalizing symptoms have been identified as a risk factor for aggression [36]. The possibility of remaining unidentified or anonymous in online communication makes aggressive behavior safer for perpetrators. This may increase the likelihood of engaging in aggressive behavior online for those suffering from internalizing symptoms [27]. Indeed, internalizing symptoms are related to cyberbullying and cyberaggression [17, 18, 27].

### Group behavior and online aggression

Social homophily is a tendency to form social ties with similar people or to prefer similar contacts over dissimilar ones [40, 41]. Similarity is a powerful determiner of human social networks [42] and an important predictor of relationship stability, especially for young people [43]. Individuals tend to favor people with similar attitudes and beliefs and to be intolerant and hostile toward holders of dissimilar ideologies [44, 45]. Social media makes it easy for users to search for like-minded users and select communities that fit their own attitudes [4]. In addition, social media platforms, algorithmic filtering technology and personal selection reduce the diversity of communication and information that people are exposed to online [4, 40, 46, 47]. This reduced social diversity may contribute to the formation of homophilic social aggregates, or “echo chambers,” which reinforce polarization and conflicts between different social and ideological cliques online [22, 23, 25].

Even given the possibilities for selectivity online, people are still also exposed to heterogeneous social contacts and antagonistic views [40, 48, 49]. Arguments in social media often emerge around public issues as people defend their views [50], and likeminded groups tend to respond negatively to confronting social contacts [23]. This might be particularly true among impulsive individuals and those with negative-affect-laden symptoms, which are associated with decreased affective control and mood instability [39, 51].

Group memberships are powerful determinants of perceived similarity and dissimilarity. According to social identity theory (SIT), an individual’s self-concept is partly defined by memberships in different social groups, and, thus, people strive to maintain a favorable comparison between “us” (in-group) and “them” (out-group) [52]. As a result, people tend to favor in-group members over out-group members and overestimate the similarity between themselves and in-group members as well as the dissimilarity between themselves and out-group members [53, 54, 55]. Currently, various online groups are increasingly important sources for social identification [56, 57, 58], discrimination and even dehumanization of out-groups [5, 19, 20, 59].

Social identification might not necessarily motivate out-group discrimination [60]. It has been suggested that deindividuation, which is a tendency to conceive the self and others in terms of group identity instead of unique personal identity, motivates out-group antipathies [25, 61]. When group identity is pronounced, deindividuation can induce self-stereotyping, which is conceiving oneself as a typical example of an in-group and identical with other in-group members [61, 62]. According to the social identity model of deindividuation effects (SIDE), the group stereotypical perception of the self and others is characteristic to online interaction [63, 64]. Online interaction often lacks individuating social cues; thus, people tend to perceive themselves and others in terms of salient group memberships, and their behavior is driven by the group norms [64, 65]. Self-stereotyping and conformity to emergent group norms can make hostile online behavior more prevalent [21, 27, 66, 67, 68].

### Research overview

We approached online hate offending from an integrative perspective, including both personal risk factors and group behavior online. As personal risk factors we analyze internalizing symptoms and impulsivity. Both of these factors have been linked to the risk of increased aggression in earlier studies [e.g. 36]). Considered forms of group behavior involve homophily, social identification, self-stereotyping and norm conformity. Previous studies have suggested both homophilic social cliques [e.g. 23, 25] and social identification with online groups [e.g. 19, 20] as predictors of aggressive online behavior. However, social identification per se might not be related to online hate offending. According to earlier studies, it may be the perception of oneself and others in terms of group stereotypes (i.e. self-categorization) and conformity to group norms in online interaction that is associated with hostile behavior [20, 25]. In this case, social identification facilitates group stereotypic behavior instead of directly inducing outgroup hostility.

In Study 1, we used a socio-demographically balanced sample of Finnish young people to analyze whether impulsivity and internalizing symptoms as personal risk factors and social homophily and social identification as forms of online group behavior predicted online hate offending. We also tested whether personal risk factors modified the association between social homophily and online hate offending. In Study 2, we conducted a survey vignette experiment to assess individual tendency to self-stereotyping and norm conformity in anonymous online interaction (with and without salient group identity) and whether they were more typical for online hate offenders than nonoffenders. In the vignette experiment, we simulated minimalistic and anonymous online interaction scenarios and then measured respondents’ self-stereotyping and group norm conformity.

## Study 1

### Method

#### Participants

The data consist of a survey collected from Finnish adolescents and young adults (*N* = 1200) in March–April 2017. The respondents were aged 15 to 25 (*M* = 21.29, *SD* = 2.85), and 50% of them were females. The demographically balanced sample mirrors the Finnish population regionally and in terms of age and gender distribution. The sample size is sufficient to detect potentially small effect sizes of interaction terms. The participant recruitment utilized the respondent panels of Survey Sampling International (SSI). Participation in the study was based on informed consent. As all the participants were 15 years-old or older, no parental consent was required. This is line with the ethical principles of the Finnish National Advisory Board on Research Ethics [69]. The Ethics Committee of the Tampere region approved the research proposal in December 2016, and the committee stated that the research did not pose any ethical problems (decision 62/2016). Respondents were contacted via email and provided with a link to an online survey. The survey was designed to study online behavior from a social psychological perspective. In this study, items concerning online hate offending and its hypothesized predictors were included in the analyses. The dataset is available on the Finnish Social Science Data Archive (see YouGamble 2017 Finland, http://urn.fi/urn:nbn:fi:fsd:T-FSD3399).

#### Measures

*Online hate offending*. Respondents were asked how often they send messages in social media that “offend or threaten other users” [for similar operationalization of online hate, see, e.g., 6, 7], with the following reply options: 1 = *never*, 2 = *less than once a year*, 3 = *at least once a year*, 4 = *at least once a month*, 5 = *more than once a month*, 6 = *once a week*, and 7 = *daily*. The distribution of our measure was highly skewed with a majority of respondents reporting never having been engaged in online hate offending (*n* = 936, 78%) and only 8% (*n* = 10) reporting online offending more than once a week or on a daily basis.

*Independent Variables*. ***Impulsivity*** was measured with the Eysenck Impulsivity Scale (EIS) [70, 71]. It consists of the following five items: “Do you generally do and say things without stopping to think?”; “Do you often get into trouble because you do things without thinking?”; “Are you an impulsive person?”; “Do you usually think carefully before doing anything?”; and “Do you mostly speak before thinking things out?” (0 = *no* and 1 = *yes*). The scale showed acceptable internal consistency (Cronbach’s α = .74). For our analysis, all items were summed up to a composite variable with higher figure indicating higher impulsivity. The composite variable was standardized for further analyses.

**Internalizing symptoms** were measured with the General Health Questionnaire (GHQ-12). GHQ-12 is a widely used instrument in screening internalizing symptoms such as anxiety and depression in general population [72, 73, 74]. The scale consists of 12 statements concerning subjective assessment of one’s mental health (e.g. *“Have you recently lost much sleep over worry*?*”* and *“Have you recently felt constantly under strain*?*”*) with four response options (e.g., *more than usual*, *same as usual*, *less than usual*, *a lot less than usual*) ordered in a manner that the bigger number always indicates worst mental health. The scale showed good reliability with a Cronbach’s alpha coefficient of .88. In accordance with the ordinal coding method for GHQ-12, the final variable for our analysis was conducted by summing up all 12 items [75] and then standardized.

**Social identification online** was measured with a cross-nationally validated online social identification scale (social identification subscale from the Identity bubble reinforcement scale, IBRS-6) [76]. The two items in which respondents were asked to assess how well the following phrases described them: “In social media, I belong to a community or communities that are an important part of my identity” and “In social media, I belong to a community or communities that I’m proud of.” For both items, the response scale ranged from 1 to 10 (1 = *does not describe me at all* and 10 = *describes me completely*). These items are based on previous social psychological operationalizations of social identification [62, 76, 77, 78]. Items had a good internal consistency (Pearson's r = .72), and, thus, they were summed up to create a count variable for further analysis. The composite variable was also standardized for further analyses.

**Social homophily online** was measured with a cross-nationally validated social media homophily scale (homophily subscale of the IBRS-6) [76] that is based on established measurement of offline homophily [79, 80]. Instead of measuring the structure of one’s social media networks, this homophily scale measures individual preference for similar-minded online interaction. The two items are: “In social media, I prefer interacting with people who are like me” and “In social media, I prefer interacting with people who share similar interests with me.” Here, again, respondents were asked to assess how well the two phrases described them on a scale from 1 to 10 (1 = *does not describe me at all* and 10 = *describes me completely*). These items were summed together to a scale (Pearson's r = .61) and the variable was then standardized.

**Covariates** included the age and gender (0 = *male*, 1 = *female*) of respondents and their social media use. In the case of social media use, respondents were asked how often they used Facebook, YouTube, Twitter, Instagram, and Instant messaging apps such as WhatsApp (0 = *do not use*, 1 = *less than once a day*, 2 = *daily*, 3 = *several times a day*).

#### Data analysis

To describe our data, we counted mean values, standard deviations, and intercorrelations for our variables (Table 1). Least squares regression models were conducted to assess the associations between online hate offending and our predictors and covariates. Due to the heteroscedasticity of residuals, we estimated robust (Huber-White) standard errors for our models. Our models with regression coefficients, standard errors, t statistics, p-values, standardized regression coefficients, and R-squared coefficients are reported in Table 2. Our analysis proceeded in two steps. Model 1 included all our predictor variables and covariates. In Model 2, interactions between our personal risk factor variables, impulsivity and internalizing symptoms, and social homophily online were added. To elaborate the significant interactions of Model 2, we plotted the change in online hate offending caused by a one-unit increase in homophily. This was done by counting average marginal effects for homophily with different values of the moderating variable.

**Table 1:** Means, standard deviations, and intercorrelations among Study 1 variables.

	M	SD	1	2	3	4	5	6	7	8	9	10	11	12
1. Online hate offending^a^	0.59	1.31	1.00											
2. Impulsivity^b^	2.32	1.36	.13***	1.00										
3. Internalizing symptoms^c^	14.15	6.35	.07*	.10***	1.00									
4. Social homophily^d^	9.09	4.2	.01	.01	-.02	1.00								
5. Social identification^d^	10.6	4.92	.12***	-.03	.05	.35***	1.00							
6. Age^e^	21.29	2.85	-.06*	-.10***	.07*	-.07**	.01	1.00						
7. Female^f^	0.50	0.50	-.22***	-.04	.24***	.05	-.02	.04	1.00					
8. Facebook use^g^	1.98	1.04	-.13***	.05	.10***	.23***	.01	.18***	.26***	1.00				
9. YouTube use^g^	2.12	0.79	.00	.01	.02	.10***	.06*	-.20***	-.24***	-.04	1.00			
10. Twitter use^g^	0.70	0.93	.10***	-.06*	-.00	.18***	.11***	-.07*	-.14***	-.01	.29***	1.00		
11. Instagram use^g^	1.63	1.19	.00	.07*	.03	.23***	.04	-.18***	.20***	.28***	.05	.12***	1.00	
12. instant messaging use^g^	2.45	0.90	-.14***	-.00	.01	.20***	.03	-.15***	.14***	.29***	.13***	.04	0.41***	1.00

^*a*^Values from 0 to 7.

^b^Values from 0 to 5 before standardization.

^c^Values from 0 to 36 before standardization.

^d^Values from 2 to 20 before standardization.

^e^Values from 15 to 25.

^*f*^*0* = male, *1* = female.

^*g*^Values from 0 to 3.

*p < .05

**p < .01

***p < 0.001.

**Table 2:** Least squares models predicting online hate offending.

	Model 1					Model 2				
	*b*	SE	*t*	*p*	*β*	*b*	SE	*t*	*p*	*β*
Impulsivity	.14	.04	3.68	**< .001**	.11	.14	.04	3.76	**< .001**	.11
Internalizing symptoms	.16	.04	4.03	**< .001**	.12	.16	.04	4.03	**< .001**	.12
Social identification	-.01	.04	-.21	.835	-.01	-.00	.04	-.09	.917	-.00
Social homophily	.14	.04	3.89	**< .001**	.11	.15	.04	4.01	**< .001**	.11
Age	-.02	.01	-1.78	.075	-.05	-.02	.01	-1.79	.073	-.05
Female	-.60	.08	-7.85	**< .001**	-.23	-.59	.08	-7.70	**< .001**	-.23
Facebook use	-.08	.04	-1.91	.056	-.06	-.08	.04	-1.88	.061	-.06
YouTube use	-.14	.05	-2.65	**.008**	-.08	-.14	.05	-2.68	**.007**	-.08
Twitter use	.11	.04	2.53	**.012**	.08	.11	.04	2.46	**.014**	.07
Instagram use	.10	.03	2.79	**.005**	.09	.09	.03	2.69	**.007**	.08
Instant messaging use	-.19	.05	-3.72	**< .001**	-.13	-.18	.05	-3.58	**< .001**	-.12
Soc. homoph. X Impulsivity						.01	.04	0.26	.798	.01
Soc. homoph. X Int. sym.						.10	.04	2.83	**.005**	.08
Constant	2.05	.34	5.99	**< .001**		2.03	.34	5.98	**< .001**	
Adjusted R^2^					.12					.12

*Soc*. *homoph* = social homophily. *Int*. *sym*. = internalizing symptoms. *b* = regression coefficient. SE = standard error. *t* = t test statistic. *p* = p value. *β* = standardized regression coefficient. Boldface indicates *p* < .05.

### Results

In our first regression model (Table 2), impulsivity (*β* = .11, *t* = 3.68, *p* < .001), internalizing symptoms (*β* = .12, *t* = 4.03, *p* < .001), and social homophily online (*β* = .11, *t* = 3.89, *p* < .001) were positively associated with online hate offending. Social identification was not associated with online hate offending (*β* = -.01, *t* = -0.21, *p* < .835). Of our covariates, online hate was negatively associated with the female gender (*β* = -.23, *t* = 7.85, *p* < .001) and the use of YouTube (*β* = -.08, *t* = -2.65, *p* = .008), and instant messaging apps (*β* = -.13, *t* = -3.72, *p* < .001). Twitter use (*β* = .08, *t* = -2.53, *p* = .012) and Instagram use (*β* = .09, *t* = -2.79, *p* = .005) were positively associated with online hate offending.

**Fig 1:**
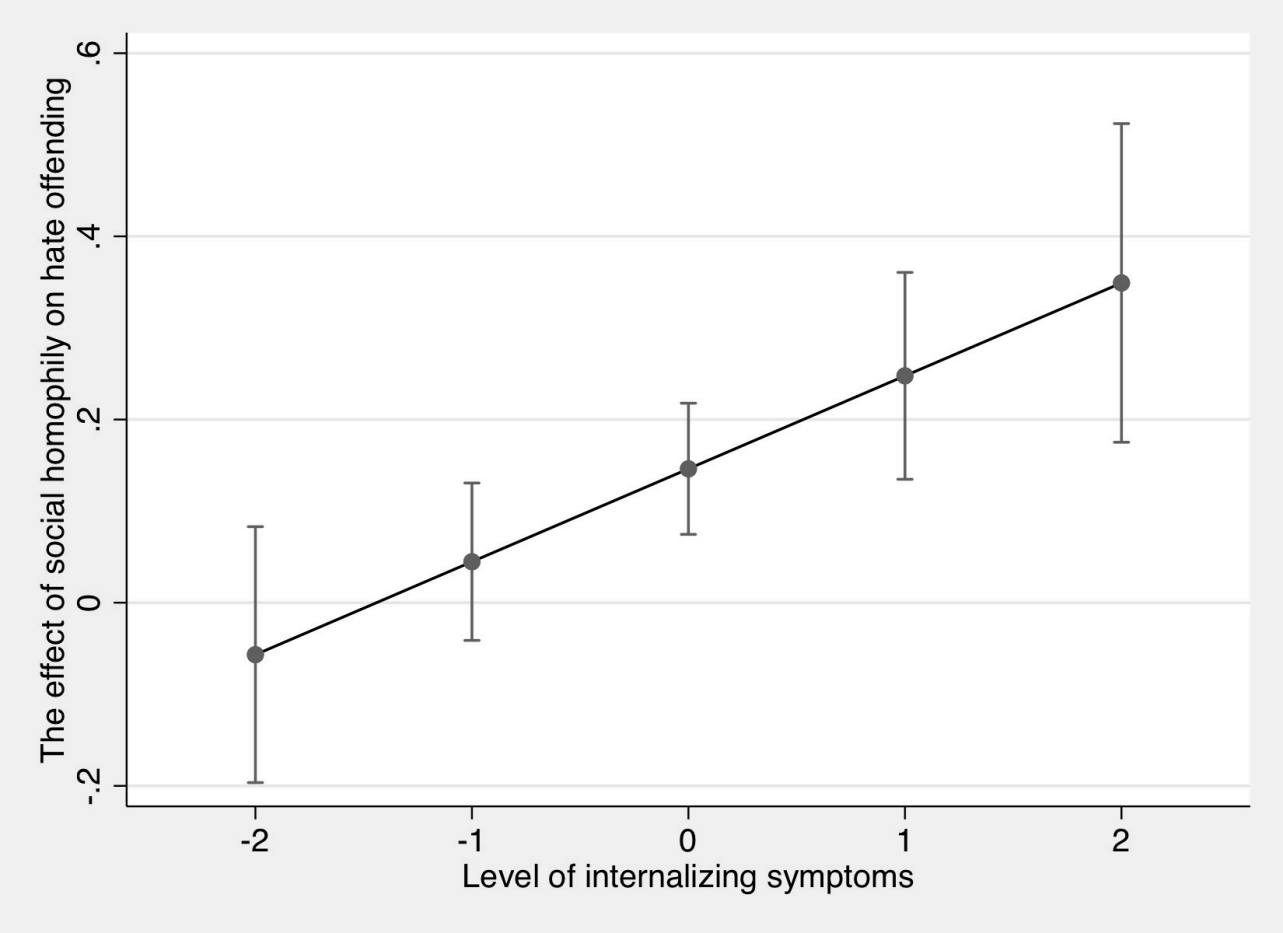
Average marginal effect of social homophily on online hate offending for different levels of internalizing symptoms. The effect refers to the change in online hate offending caused by a one-unit increase in homophily.

In our second regression model, internalizing symptoms moderated the association between social homophily and online hate offending (*β* = .08, *t* = 2.83, *p* = .005). According to the moderation effect, the positive association between social homophily and online hate offending only concerns those with average or high internalizing symptoms (see Fig 1). There was no significant association between online hate offending and homophily for those with low internalizing symptoms (one standard deviation below the mean or less). The interaction between impulsivity and social homophily online was not significant (*β* = .01, *t* = 0.26, *p* = .798).

## Study 2

In Study 1, social identification was not associated with online hate offending. To elaborate the possible connection between social identity and online hate offending, we conducted a vignette experiment with simulated anonymous and minimalistic online interaction scenarios. In this Study 2, we analyzed whether online hate offenders are more likely to perceive themselves in terms of group stereotypes (i.e. self-stereotype) in online communication if shared group identity is either primed or absent. We also tested whether online hate offenders were more likely to conform to a perceived group norm. To test these associations, we used an experimental design to measure the tendency to self-stereotype in online interaction [for a similar approach on prejudice, see 81].

### Method

#### Participants and procedure

The data consist of an online survey, including a vignette experiment, collected in April–June 2017. The sample size (*N* = 160) is sufficient to detect main effects of r = ± .22 (two-tailed α = .05 and β = .20). Participants were Finnish adolescents and young adults aged 15 to 25 (*M* = 22.48, *SD* = 2.58), and 57% (*n* = 91) of them were females. Respondents were recruited via Finnish discussion forums and social networking sites, and they were provided with a link to an online survey. For compensation, respondents had an opportunity to take part in a movie ticket draw. The study was conducted according to the ethical principles of the Finnish National Advisory Board on Research Ethics [69]. The Ethics Committee of the Tampere region approved the research proposal in December 2016, and the committee stated that the research did not pose any ethical problems (decision 62/2016). The dataset is available on the Finnish Social Science Data Archive (see YouGamble 2017 Finland, Social Media, http://urn.fi/urn:nbn:fi:fsd:T-FSD3400).

First, the survey targeted relevant background questions on social media use and online behavior (similar to Study 1). After this, the survey included a vignette experiment using a mixed design with one 2-level between-subject and 2 x 2 x 2 within-subject factorial design. In the beginning of the experimental section, respondents were randomly assigned into one of the two conditions (the 2-level between-subject design): a salient group identity condition or a control condition for the experiment. In the salient group identity condition, respondents were told: “**You have been placed in group C**, because your answers have been similar to the answers of the other group members.” Those in the control condition were given no group information.

Next, all respondents were shown social media messages on gambling. They were then asked to indicate whether, in a real social media setting, they would “like” (thumbs up) or “dislike” (thumbs down) the message or whether they would not react to the message at all. For each message, we manipulated the majority opinion, the stance towards gambling and the used narration in the message (the 2 x 2 x 2 within-subjects design). *Majority opinion* was manipulated by showing that, in half of the vignettes, a majority (about 85%) had “disliked” the message and, in the other half, the majority had “liked” the message. For those in the salient identity condition, the distribution of “likes” and “dislikes” was framed as their in-group members’ earlier responses. The *stance towards gambling* was manipulated by showing half of the messages as pro-gambling oriented (discussed the benefits of gambling, e.g., entertainment value), and the other half as anti-gambling oriented (discussed gambling-related harms, e.g., gambling problems). The third manipulated factor was *the used narration* of the message. Half of the messages were narrated as experience based (first-person narration, e.g., one’s own gambling experiences), while the other half was narrated as fact based (third-person narration, e.g., research findings on gambling). For exact manipulations, see the English translated vignette messages in S1 Appendix.

The combined between-subject design (2x) and within-subject design (2 x 2 x 2) resulted in eight different vignette scenarios for both group condition and control condition (16 in total. For both conditions, the vignettes were partitioned into two vignette sets (with four vignettes each) which were then randomly assigned to the respondents. The factorial structure of the vignette sets was designed in a manner where both pro-gambling and anti-gambling content and experience-based and fact-based narration were depicted as “liked” by the majority on one occasion [82]. Thus, the group norm did not “favor” any form of gambling orientation or narration.

The experiment described above was originally designed to study how young people react to gambling content in social media (the preregistered hypotheses can be found at https://osf.io/m72hz/) [see also 83]. In this study, we utilize the experiment to analyze whether self-reported online hate offending is associated with self-stereotyping in anonymous online interaction and conformity to perceived group norms.

#### Measures

**The absence of group identity** was added in the analysis as a dichotomous measure (0 = for *group identity condition* and 1 = *for control condition*).

**Self-stereotyping** was measured with two items on a scale from 1–10 (1 = *strongly disagree* and 10 = *strongly agree*): “I have a lot in common with the other group members/respondents” and “I am similar to the other group members/respondents” [adapted from 62]. These questions were presented to respondents after they had completed all four vignettes. As the measure consisted of only two questions, and they showed good internal consistency (Pearson's r = .86), the items were summed up and used as an observed variable in our path analysis.

**Norm conformity** was calculated as a sum of occasions when the respondent followed the perceived group norm (i.e. the majority) in his or her reactions. That is, we summed up dislikes in those vignettes where the majority had disliked the content, and likes in those vignettes where the majority had liked the content [see 81, 82]. This resulted in a variable with a range from 0 (did not follow the group norm once) to 4 (followed the group norm every time).

**Online hate offending** was measured with the same question posed in Study 1 concerning online hate offending (1 = *never*, 2 = *less than once a year*, 3 = *at least once a year*, 4 = *at least once a month*, 5 = *more than once a month*, 6 = *once a week*, and 7 = *daily)*. This question was presented after the experiment and the measure was used as an exogenous variable in our path model.

#### Data analysis

**Fig 2:**
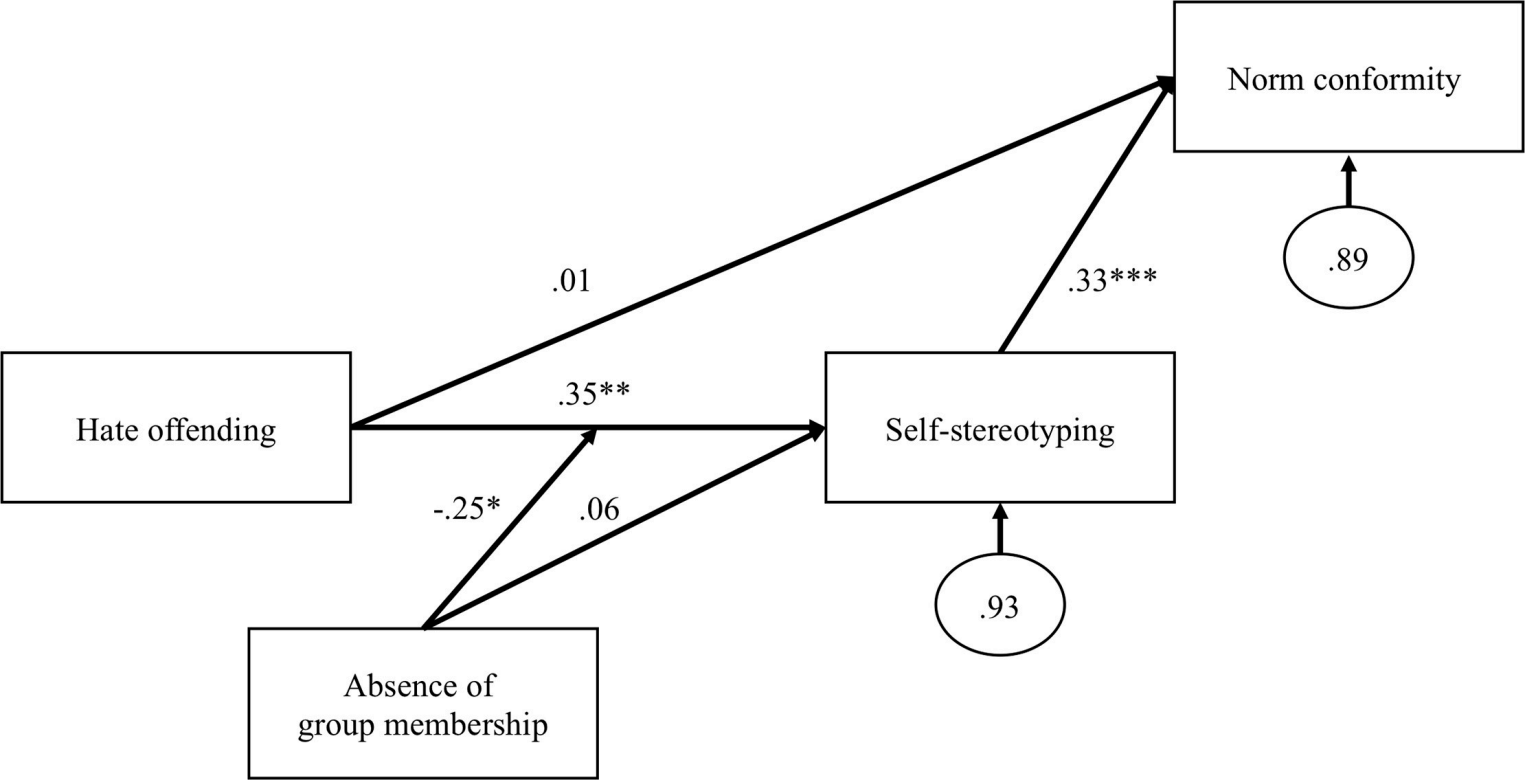
The path model in Study 2 with standardized regression coefficients. *p < .05, **p < .01, *** p < .001.

Descriptive statistics with mean values, standard deviations, and intercorrelations among the Study 2 variables are reported in Table 3. We used structural equation modelling with maximum likelihood estimation to conduct path model analysis. Satorra–Bentler adjustments were used to account for the heteroskedasticity of residuals. Our path model, including beta coefficients and their statistical significance, is reported in Fig 2.

**Table 3:** Means, standard deviations, and intercorrelations among Study 2 variables.

	M	SD	1	2	3	4
1. Online hate offending^a^	0.54	1.20	1.00			
2. Identity salience^b^	0.47	0.50	-.01	1.00		
3. Self-stereotyping^c^	7.24	3.85	.14*	-.01	1.00	
4. Norm conformity^d^	0.21	0.74	.07	.02	.34**	1.00

^a^Values from 0 to 7.

^*b*^*0* = control condition, *1* = salient group identity condition.

^c^Values from 2 to 18.

^d^Values from 0 to 4.

*p < .05

**p < .01

***p < 0.001.

In addition, we report in the text fit statistics for our model (χ^2^(2), CFI, RMSEA with 90% CI, and SRMR) as well as beta coefficient and statistical significance estimates for the indirect association between hate offending and conformity to perceived group norm (via self-stereotyping).

As our path model includes moderation, we further analyzed our statistical power to detect interactions of similar effect size in replicated studies using same sample size (with α = 0.05). To achieve this, we bootstrapped our analysis 10,000 times and report the proportion of replications that were able to reject the null hypothesis (i.e. there is no significant moderation effect) [see e.g. 84].

### Results

There was a good fit between our model and the data: scaled *χ*^*2*^*(7)* = 27.54, *CFI* = 1.000, *RMSEA* = 0.000, *90% CI* [< .001, .108], *SRMR* = .013. In our model (Fig 2), hate offending was associated with self-stereotyping (*ß* = .35, 95% CI .13, .57], *z* = 3.13, *p* = .002) in the case of salient group membership. The interaction term between hate offending and absence of group membership was significant as well (*ß* = -.25, 95% CI -.48, -.03], *z* = 2.22, *p* = .026). Online hate offending was not associated with self-stereotyping when group membership was absent (*ß* = -.01, 95% CI [-.21, .20], *z* = 0.05, *p* = .959). According to our power analysis, our power to detect interaction terms with given effect size was .65 (α = .05, two-tailed test). In our bootstrapped replications, we were able to reject the false null hypothesis 6,547 times out of 10,000 samples (with N = 160).

Self-stereotyping had a positive association with conformity to group norms (*ß* = .33, 95% CI [.19, .47], *z* = 4.68, *p* < .001). There was no significant direct association between online hate offending and conformity to group norms (*ß* = .01, 95% CI [-.12, .14], *z* = 0.14, *p* = .891). However, there was a significant indirect association between online hate offending and conformity to group norms via self-stereotyping in the case of salient group membership (*ß* = .11, *z* = 2.56, *p* = .011) but not without a shared group identity (*ß* = -.00, *z* = 0.05, *p* = .959).

### Discussion

In two studies, we analyzed online hate offending by using an integrative approach including both personal risk factors and online group behavior. Of our personal risk factors, both impulsivity and internalizing symptoms were associated with more likely online hate offending. In line with earlier research on impulsivity and aggressive behavior [36] and online aggression [15, 16, 27], it appears that impulsive individuals are more likely to offend or threaten others online. Impulsive individuals show less self-reflection or hesitation in their online communication and, thus, they might fail more often than others to inhibit their behavior or “think before they post” [11]. Impulsivity is also related to personality disorders [35], and it is possible that the association between impulsivity and hate offending is partly explained by antisocial personality traits.

Online hate offending was also positively associated with internalizing symptoms. Internalizing symptoms may cause intensive negative affective states and dysfunctional emotional and behavioral regulation. Dysregulated behavior can be a way to distract oneself from these intensive negative affective states [39]. Consequently, internalizing symptoms can be related to aggressive behavior [36], especially on social media where such behavior may be safer for perpetrators (e.g. due to possibilities of anonymity or lack of physical contact with the victims) [27].

In addition to personal risk factors, we found that online hate offending was associated with online group behavior. This is in line with earlier literature on cyberaggression. Echo chambers of likeminded individuals [22, 23, 25] and social identification with online groups [20, 59] are reported to induce hostilities between fragmented ideological groups, and deindividuated group-norm-driven online behavior is likely to reinforce antisocial behavior [63, 66, 68].

More than others, online hate perpetrators preferred social ties who share their interests or are similar to them in other ways. Thus, it is likely that online hate offending occurs in situations where individuals who prefer attitudinal homophily are exposed to opposing views. This is in line with earlier research suggesting that the exposure to opposing groups or attitude-challenging information often provokes arguments and negative responses online [23, 50]. In addition, like-minded social media cliques (i.e. echo chambers) reinforce attitude polarization [22]. This effect might be particularly strong among those individuals with high preference for attitudinal homophily.

However, preference for homophily was only associated with more likely online hate offending among individuals with average or high negative-affect-related internalizing problems. In other words, people with low internalizing symptoms seem to tolerate dissimilarity rather well, as social homophily was not associated with online hate offending among them. This also implies that even high preference for similarity (or low tolerance for different opinions) might not motivate aggression in online interaction in the absence of relevant personal risk factors (here, reduced psychological wellbeing).

According to our findings, online hate offenders do not identify with online communities more than others, but they are more likely to use group stereotypes and follow group norms in anonymous online interaction. This mirrors earlier studies suggesting that social identification might not always encourage negative attitudes towards other groups [60]. Social identification online may mainly be a positive factor that encourages participation and engagement in various online groups [58]. However, social identification was found to facilitate self-stereotyping among online hate offenders. That is, hate offenders were more likely to self-stereotype in simulated online interaction scenarios with a shared group identity. This association did not exist when common group identity was not primed. Furthermore, online hate offending was positively associated with group norm conformity but only indirectly via self-stereotyping.

Our findings suggest that online social identities become a resource for hostile behavior when people start making distinctions between similar “us” and dissimilar “others” or conceive online interaction mainly in terms of deindividuated group memberships. Online communication facilitates interaction where people see themselves and others in terms of groups rather than individual identities [63, 66, 68]. Thus, in the case of deindividuated group offending, threatening or offending content is not posted from one individual online user to another but by deindividuated group members to deindividuated targets [25].

### Limitations

Self-reporting of socially sanctioned and potentially illegal behavior, such as online hate, can suffer from so called social desirability bias that leads to under reporting of such behavior [83, 84]. Nonetheless, according to methodological inquiry, this bias can be reduced with an appropriate survey design. The primary methods for this include the use of anonymous self-administrated surveys that involve no social interaction with an interviewer and explicit assurances of confidentiality to survey respondents [85, 86]. Both of these strategies were utilized in this research to reduce motivation for biased reporting, such as the need for social approval, self-presentation concerns and impression management [85].

Self-reported measures are also limited by other factors. For instance, the respondents might have simply forgotten their earlier aggressive interaction episodes or not have interpreted them as hostile in the first place. It is also possible that individual differences in self-reporting are related to personal characteristics and background factors such as internalizing symptoms, impulsivity, age or gender. These limitations of self-report measures should be acknowledged when interpreting the results of this study.

As our samples were not based on probability sampling, the potential issues of representativeness should be acknowledged when interpreting our findings. However, our Study 1 sample was demographically well balanced in terms of gender, age, and living area. The convenience sample used in Study 2 was relatively small. In order to achieve better statistical power, we recommend that future studies use larger samples. In addition, our samples only consisted of Finnish respondents. There is a need for studies testing whether our findings apply to other national and cultural contexts.

Furthermore, our analysis was mainly based on cross-sectional data, and, thus, it does not allow for straightforward causal inference. The direction of found associations was interpreted on the basis of our theoretical framework. In Study 2, we complemented our cross-sectional approach with a vignette experiment to isolate personal tendency to self-stereotype and follow emergent group norms and assessed their relationship with self-reported online hate offending. There are still limitations for the causal inference, as we did not measure the online hate offending in the experiment but relied on the self-report measure of previous offending. However, this approach of combining the pre-experiment measurement and minimal group experiment has been used in previous research to examine the relationship between social identity dynamics and prejudice [81].

Our vignette scenarios only involved minimalistic interaction with the group (distributional information of others’ reactions) and restricted binary reactions (likes or dislikes). However, even simple forms of interaction and shared binary reactions have been found to facilitate social identification processes and norm construction in classic experiments [52] and in online interaction [57, 87]. Our results are in line with these studies. It should be noted as well, that our experimental vignettes were gambling-related. However, the group norms within the vignette scenarios did not favor any stance towards gambling such as critical or positive gambling attitudes.

### Conclusion

In our studies with integrative social psychological framework, we found that both personal risk factors and online group behavior are associated with online hate offending. Personal risk factor related online hate was expressed by people who are less able to inhibit their behavior or self-reflect their actions online or had reduced psychological wellbeing. Group behavior-related online hate is related to categorizing the self and others in groups of similar us and dissimilar others and by framing online interaction in terms of deindividuated group stereotypes. This group behavior-related online hate may be reinforced by personal risk factors such as internalizing symptoms.

Recognizing these two types of explanations is important, as they have different implications for reducing hateful communication online. Interventions fostering reflection and self-monitoring in online interaction could be effective for impulsive online hate offending [11]. For group behavior-related offending, the most effective measures could be those involving the enhancement of social and ideological diversity [47] or prosocial group norms [65] in an online space. Future research should further scrutinize the effectivity of different online interventions in tackling these two types of online hate.

## Supporting information

S1 Appendix English-translated vignettes and manipulations used in the survey experiment. (DOCX)
