# Supplementary material for: Impulsivity, internalizing symptoms, and online group behavior as determinants of online hate
Source: PLoS One. 2020 Apr 22;15(4):e0231052. doi: 10.1371/journal.pone.0231052 (PMC7176079; doi:10.1371/journal.pone.0231052)
Supplement: S1 Appendix — (DOCX) [file pone.0231052.s001.docx]

Appendix 1: *English-Translated Vignettes and Manipulations Used in the Survey Experiment*

| Condition | Vignette |
| --- | --- |
| Experience-driven, pro-gambling | *Me and many of my friends* **gamble**. Gambling **brings** ***me enjoyment***, and it ***has*** ***brought significant benefits*** *to me and my family’s well-being*. Behind the following link, you can read more *about Americans’ experiences* on gambling. |
| Fact-driven, pro-gambling | *According to a recent report, 77% of Americans* **gamble**. Gambling **brings *enjoyment*,** and it ***brings significant benefits*** ***to*** *the society and people’s well-being*. Behind the following link, you can read more *research findings* on gambling. |
| Experience-driven, anti-gambling | *Me and many of my friends* **suffer from gambling problems**. Gambling **causes *me problems***, and it ***has caused significant damage*** *to me and my family’s well-being*. Behind the following link, you can read more *about Americans’ experiences* on gambling. |
| Fact-driven, anti-gambling | *According to a recent report, more than 5 million Americans* **suffer from gambling problems**. Gambling **causes *problems***, and it ***causes significant damage for*** *the society and people’s well-being*. Behind the following link, you can read more *research findings* on gambling. |

*Note*. Italics indicate fact-driven / experience-driven manipulations. Bold font indicates pro-gambling / anti-gambling manipulations.
